# Four-week short chain fructo-oligosaccharides ingestion leads to increasing fecal bifidobacteria and cholesterol excretion in healthy elderly volunteers

**DOI:** 10.1186/1475-2891-6-42

**Published:** 2007-12-05

**Authors:** Yoram Bouhnik, Lotfi Achour, Damien Paineau, Michel Riottot, Alain Attar, Francis Bornet

**Affiliations:** 1 Pôle des Maladies de l'Appareil Digestif (PMAD), Service de Gastroentérologie et d'Assistance Nutritive, Hôpital Beaujon, 100 bd du Général Leclerc, 92110 Clichy Cedex, France; 2 Institut Supérieur de Biotechnologie, avenue Taher El HADED, BP.74, 5000 Monastir, Tunisia; 3 Nutri-Health SA, Immeuble Ampère, 8 rue Eugène et Armand Peugeot, 92566 Rueil-Malmaison Cedex, France; 4 Université Paris Sud, Laboratoire de Physiologie de la Nutrition, Bat. 447, 91405 ORSAY Cedex, France

## Abstract

**Background:**

Short-chain fructo-oligosaccharides (scFOS) are increasingly used in human diet for their prebiotic properties. We aimed at investigating the effects of scFOS ingestion on the colonic microflora and oro-fecal transit time in elderly healthy humans.

**Methods:**

Stools composition, oro-fecal transit time, and clinical tolerance were evaluated in 12 healthy volunteers, aged 69 ± 2 yrs, in three consecutive periods: basal period (2 weeks), scFOS (Actilight^®^) ingestion period (8 g/d for 4 weeks) and follow-up period (4 weeks). Two-way ANOVA, with time and treatment as factors, was used to compare the main outcome measures between the three periods.

**Results:**

Fecal bifidobacteria counts were significantly increased during the scFOS period (9.17 ± 0.17 log cfu/g vs 8.52 ± 0.26 log cfu/g during the basal period) and returned to their initial values at the end of follow-up (8.37 ± 0.21 log cfu/g; P < 0.05). Fecal cholesterol concentration increased during the scFOS period (8.18 ± 2.37 mg/g dry matter vs 2.81 ± 0.94 mg/g dry matter during the basal period) and returned to the baseline value at the end of follow-up (2.87 ± 0.44 mg/g dry matter; P < 0.05). Fecal pH tended to decrease during scFOS ingestion and follow-up periods compared to the basal period (P = 0.06). Fecal bile acids, stool weight, water percentage, and oro-fecal transit time did not change throughout the study. Excess flatus and bloating were significantly more frequent during scFOS ingestion when compared to the basal period (P < 0.05), but the intensity of these symptoms was very mild.

**Conclusion:**

Four-week 8 g/d scFOS ingestion is well tolerated and leads to a significant increase in fecal bifidobacteria in healthy elderly subjects. Whether the change in cholesterol metabolism found in our study could exert a beneficial action warrants further studies.

## Background

Short-chain fructo-oligosaccharides (scFOS) are a mixture of oligosaccharides consisting of glucose linked to fructose units [1]. They are poorly absorbed in the human small intestine [2], but are fermented in the colon by the resident microflora [3]. It is now well established that scFOS meet criteria to be considered as prebiotic, defined as a non digestible food ingredient that beneficially affects the host by selectively stimulating growth and/or activity of one or a limited number of colonic bacteria, and thus improves host health [4]. We have shown in humans that dietary addition of 10 g/d scFOS led to increasing fecal counts of bifidobacteria [5]; moreover, the scFOS administration dose-dependently increases fecal bifidobacteria in healthy volunteers, with an optimal and well-tolerated dose ranging from 2.5 to 10 g/d [6,7].

Bifidobacteria are considered beneficial to health [8], even if sound evidence of such effect is not available yet [4]. In mice, *in vivo *administered bifidobacteria along with fructo-oligosaccharides reduced 1,2-dimethylhydrazine induced carcinogenesis [9]. In rats, *Bifidobacterium longum*, administered alone or in association with non-digestible oligosaccharides, exerts strong antitumour activity [10,11] This effect could be due to colon acidification that inhibits bacterial degradation of primary to carcinogenic secondary bile acids [12] and/or to increasing bifidobacteria population. Indeed, bifidobacteria *per se *could have an anti-tumorigenic activity. Bifidobacteria reduce nitrosamine mutagenicity, and *Bifidobacterium bifidum *administered along with *Lactobacillus acidophilus *to healthy humans decreases nitroreductase activity in stools [13]. Lastly, oligosaccharide ingestion could result in increasing colonic contents and decreasing transit time [14], both factors may affect colonic carcinogen concentration and mucosal contact time [15]. Thus, taking into account the intrinsic anti-tumoral properties of bifidobacteria and the effects on colonic pH, fecal mass and transit time, a potential benefit of scFOS ingestion could be colon cancer prevention, in particular in the elderly, who are particularly at risk of developing colon cancer [16].

Although colonic microbiota is relatively stable throughout adult life, age-related changes in the gastrointestinal tract inevitably affect its composition [17]. Bifidobacteria are numerically important colonic species that can be found in adults [18], and the decline in bifidobacteria numbers is one of the most marked changes in the elderly gut [19]. These changes, along with general reduction in species diversity in most bacterial groups, as well as changes in diet and digestive physiology, such as intestinal transit time, may result in increased putrefaction in the colon, and greater susceptibility to disease. Dietary supplements containing prebiotics have been suggested to counteract these changes in the elderly [20-22].

In that context, the aim of our study was to assess in healthy elderly the effects of four-week scFOS ingestion on colonic microflora and oro-fecal transit time (OFTT).

## Methods

### Subjects

Twelve elderly healthy volunteers, six men and six women, aged 69 ± 2 years, participated in the study. None of them had any gastrointestinal disease history. No antibiotics or laxatives had been taken during the 3 months before the study. No other medication was allowed during the investigation period. The subjects signed a written informed consent to the protocol, which was approved by Lariboisière – Saint-Louis Hospital Ethics Committee.

### Study Design

The study was conducted in Saint-Lazare Hospital, Paris, France. It was divided into three periods: basal (weeks 1–2), scFOS (weeks 3–6), and follow-up (weeks 7–10) periods. Throughout the study, volunteers took their usual diet. Neither fermented dairy products containing viable bifidobacteria and FOS (onions, asparagus, rye, and Jerusalem artichoke) were allowed, nor food known to induce abdominal symptoms (beans, cabbage, raisin, banana, and wheat bran). During scFOS period, subjects received 8 g/d scFOS in two oral doses at the end of breakfast and diner. This dose was defined as a good compromise between efficacy and tolerance. We used scFOS from Actilight^® ^(Beghin Meiji, Marckolsheim, France), which consist of 44% 1-ketose (GF_2_), 46% nystose (GF_3_), and 10% 1^F^-β-fructofuranosyl nystose (GF_4_).

To measure the mean oro-fecal transit time (OFTT), the subjects ingested, with their breakfast, 20 radio-opaque pellets of different shapes for three consecutive days [23]. The first stools passed after the fourth day were collected, and their marker content analysed. Stools had been collected for three consecutive days before the end of weeks 2 (basal period), 6 (ingestion period), and 10 (follow-up period), that is to say at the end of each feeding period.

Tolerance to administered scFOS was evaluated using a daily chart in which the symptoms (excessive flatus, borborygmi, bloating, and abdominal pain) were rated from zero (no symptom) to three (severe symptom). Stool frequency and consistency were also graded by the volunteers. Diarrhoea was defined as one or more watery stools, or more than three stools per day.

### Stool collection

Stools were collected three times, for 48 h at the end of each period (weeks 2, 6, and 10). Samples were collected in plastic containers rendered anaerobic (Anaerocult A; Merck, Darmstadt, Germany), immediately transferred to the laboratory, and then analysed for bacterial counts and pH within 2 hours. Stools were then frozen at -20°C for further analysis.

### Bacterial counts and pH

Fresh faecal samples (1 g) were introduced in the first pre-weighed tube of the dilution series and thoroughly mixed, then further tenfold dilutions were made up to -9 in a reduced diluent (1/4 strength cysteinated Ringer diluent). 0.1 ml of each dilution was spread on plates with different selective media to distinguish several bacterial genera: total anaerobic counts (Wilkins-Chalgren agar), Bifidobacterium (Beerens' medium), Clostridium spp. (TNS medium), and enterobacteria (McConkey agar). The tests were duplicated for the first two media. Plates of the first three media were anaerobically incubated for 5 to 7 d, and McConkey agar was aerobically incubated for 48 hours. Colony counts were obtained and expressed as a log of the colony-forming units (CFU) per gram of fresh faeces. Extemporarily, the fresh stool pH was measured by pH meter (Bioblock, Illkirch, France).

### Bile acids

For bile acid and neutral sterol analysis, frozen stools had been lyophilised and lipids had been extracted with ethanol for 24 hours in a Soxhlet apparatus. Lipid fractions had been saponified in boiling ethanolic 2 m potassium hydroxide for 1 h. Sterols were extracted with hexane, and bile acids were deconjugated. [24] Total bile acids were measured by 3-hydroxy-steroid-dehydrogenase, according to slight modification of Stempfel and Sidbury technique [25]. Prior to enzyme determination, bile acids were dissolved in 2-propanol. Free bile acids were methylated with diazomethane, silylated with Deriva-sil (Chrompack, Middelburg, The Netherlands), and assayed on Carlo Erba (Milan, Italy) HGRC 5160 gas chromatograph equipped with standard fused silica WCOT capillary column cross-linked with OV1701 (Spiral, Dijon, France) (length, 25 m; film thickness, 0.2 lm; oven temperature, 240°C; hydrogen carrier gas flow rate, 2 mL/min). Faecal sterols were silylated with bis(trimethylsilyl)tri-fluoroacetamide (BSTFA) + 1% trimethylchlorosilane (TMCS) (Pierce, Rockford, IL, USA), and quantified using gas chromatography described above, with the following modifications: fused silica WCOT OV 101 capillary column (Spiral, Dijon, France) (length, 25 m; film thickness, 0.2 lm; oven temperature, 220°C).

### Data analysis

Faecal bacteria concentrations were expressed as log colony forming unit (cfu)/g wet weight. The results were expressed as means ± SEM for each period. Two-way ANOVA, with time and treatment as factors, was used to compare bacterial concentrations, pH, and faecal metabolites between the three periods. Following a significant F test (P < 0.05), Newman-Keuls test was used to identify differences between individual means. Symptoms experienced with scFOS were compared to those experienced with placebo using Wilcoxon signed rank test.

## Results

### Bacterial counts and pH

Table 1 summarises bacterial counts and pH during basal, scFOS, and follow-up periods. Faecal bifidobacteria counts were significantly increased during the scFOS period (9.17 ± 0.17 log cfu/g vs 8.52 ± 0.26 log cfu/g during the basal period; P < 0.05), and returned to their baseline values during the follow-up period (8.37 ± 0.21 log cfu/g). Total anaerobe counts did not change during scFOS period compared to the basal period, but decreased in the follow-up period compared to the ingestion period (P < 0.05). Faecal *Clostridium *counts were significantly increased during the follow-up period compared to the basal and scFOS periods (P < 0.05). Faecal enterobacteria counts did not change during the three periods. Faecal pH tended to decrease during scFOS and follow-up periods compared to the basal period (P = 0.06).

**Table 1:** Faecal bacterial counts (log cfu/g wet weight) and pH in elderly healthy volunteers during basal (2 wks), scFOS (4 wks) and follow-up (4 wks) periods (n = 12, mean ± SEM)

	**Basal period**	**scFOS period**	**Follow-up period**
**Bifidobacteria**	8.52 ± 0.26	9.17 ± 0.17^a^	8.37 ± 0.21
**Total anaerobes**	10.09 ± 0.07	10.22 ± 0.06^b^	9.94 ± 0.09
***Clostridium***	3.25 ± 0.25	3.45 ± 0.26^b^	4.29 ± 0.30^c^
***Enterobacteria***	7.69 ± 0.21	7.45 ± 0.28	7.48 ± 0.24
**PH**	6.57 ± 0.10	6.32 ± 0.10	6.26 ± 0.07

^a ^different from basal and follow-up periods (P < 0.05)

^b ^different from follow-up period (P < 0.05)

^c ^different from basal period (P < 0.05)

### Faecal neutral sterols and bile acids

**Figure 1:**
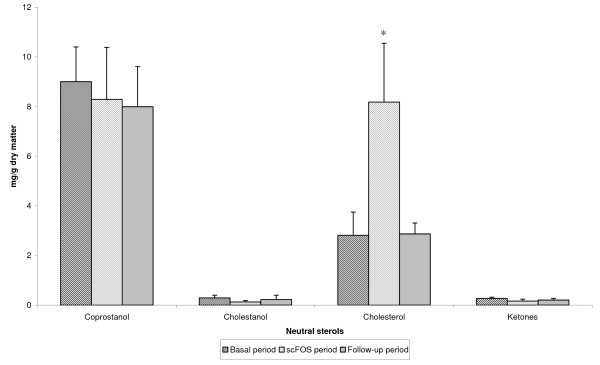
**Effect of 4-wk scFOS ingestion (8 g/d) on faecal cholesterol in healthy volunteers (means ± SEM, n = 12)**. *P < 0.05 between scFOS period and both basal and follow-up period

Faecal cholesterol concentration increased during the scFOS period (8.18 ± 2.37 mg/g dry matter vs 2.81 ± 0.94 mg/g dry matter during the basal period; P < 0.05)), and returned to the baseline value during the follow-up period (2.87 ± 0.44 mg/g dry matter (figure 1). However, no statistical differences were reported for coprostanol, cholestanol, and ketones for the three periods (Table 2). Total neutral sterol concentrations and outputs did not change, but tended to increase (p = 0.08) during the scFOS period.

**Table 2:** Faecal neutral sterols and bile acids (mg/g dry matter) in elderly healthy volunteers during the basal (2 wks), scFOS (4 wks) and follow-up (4 wks) periods (n = 12, mean ± SEM)

**Neutral sterols**	**Basal period**	**scFOS period**	**Follow-up period**
Coprostanol	9.00 ± 1.40	8.29 ± 2.09	7.99 ± 1.62
Cholestanol	0.29 ± 0.11	0,13 ± 0.05	0.23 ± 0.17
Cholesterol	2.81 ± 0.94^a^	8.18 ± 2.37^b^	2.87 ± 0.44^a^
Ketones	0.27 ± 0.05	0.16 ± 0.08	0.21 ± 0.06
Total	12.38 ± 1.15	16.75 ± 1.94	11.30 ± 1.57

**Bile acids**	**Basal period**	**scFOS period**	**Follow-up period**

Lithocholic	2.00 ± 0.43	1.29 ± 0.29	1.26 ± 0.17
Deoxycholic	1.80 ± 0.35	2.58 ± 0.50	2.61 ± 0.63
Cholic	0.46 ± 0.21	0.58 ± 0.19	0.87 ± 0.24
Chenodeoxycholic	0.24 ± 0.05	0.20 ± 0.04	0.30 ± 0.07
Ketones	1.26 ± 0.30	1.52 ± 068	1.33 ± 0.19
Total	5.77 ± 0.66	6.17 ± 1.25	6.37 ± 1.02

a ≠ b : P < 0.05

Total bile acid concentrations and outputs were similar in the three periods. Concentrations of secondary (lithocholic and deoxycholic acids) and primary bile acids (cholic and chenodeoxycholic acids) did not change for the three periods (Table 2).

### Stool weight and oro-fecal transit time

Stool wet weight, dry matter, and faecal water did not change throughout the study. Oro-fecal transit time was not significantly modified by scFOS ingestion compared to the basal and follow-up periods (Table 3).

**Table 3:** Mean oro-fecal transit time (OFTT) and mean 24-h faecal wet weight, dry weight and percentage of faecal water in elderly healthy volunteers during the basal (2 wks), scFOS (4 wks) and follow-up (4 wks) periods (n = 12, mean ± SEM)

	**Basal period**	**scFOS period**	**Follow-up period**
OFTT (h)	37.2 ± 3.4	39.9 ± 3.3	37.8 ± 3.7
Wet weight (g/d)	155.4 ± 20.9	137.7 ± 17.3	174.8 ± 22.0
Dry weight (g/d)	32.8 ± 3.3	28.8 ± 2.9	35.2 ± 3.5
Faecal water (%)	77 ± 2	77 ± 1	76 ± 2

### Digestive tolerance

During scFOS ingestion, excessive flatus and bloating were significantly more frequent when compared to the basal period (P < 0.05), but symptom intensity was very mild (Table 4). Borborygmi and abdominal pain were not significantly different in all periods.

**Table 4:** Digestive symptom intensity (ranged from 0 to 3) in elderly health volunteers during the basal (2 wks), scFOS (4 wks) and follow-up (4 wks) periods (n = 12, mean ± SEM)

	**Basal period**	**scFOS period**	**Follow-up period**
Excessive flatus	0	0,83 ± 0,3^a^	0,25 ± 0,13
Bloating	0	0,67 ± 0,26^a^	0,33 ± 0,14
Borborygmi	0	0	0
Abdominal pain	0	0,42 ± 0,23	0,25 ± 0,13

Symptom intensity was rated as follow: 0: no symptom ; 1: mild symptoms; 2: moderate symptoms; 3: severe symptoms

^a ^different from basal period (P < 0.05)

## Discussion

The present experiment showed that four-week scFOS ingestion, with a dose of 8 g/d, is well tolerated and leads to significant increase in faecal bifidobacteria and cholesterol excretion in healthy elderly. The sc-FOS bifidogenic effect has been extensively demonstrated in adults [6,26-28], but rarely in elderly [19,21,22]. Among the very few available studies about functional foods in elderly, one recent double-blind trial testing a symbiotic (B. lactis BL-01, B. bifidum BB-02 and an inulin-based prebiotic) also found promising results [29]. Significant increase in total bifidobacteria counts was indeed observed in the symbiotic group compared with the placebo group.

We also found increasing *Clostridium spp*. after sc-FOS ingestion discontinuation. *Clostridium spp*. is a major component of normal anaerobic microflora and it can not be considered as a deleterious or beneficial genus. Since some toxinogenic subspecies of *Clostridium difficile *are related to an increased risk of pseudo membranous colitis and/or infection in older people, it would have been interesting to measure the sc-FOS effects on these subspecies. However, we did not perform those analyses, for they were out of our study scope. Further studies may investigate this point, using adequate measurement methods for species concentrations and toxinogenic properties (cellular cultures, biomolecular analysis). Culture-based enumeration of microbiota does not usually allow for bacterial species measurement, but mainly bacterial genus.

In our study, several parameters were assessed with the objective to better understand scFOS physiological effects in healthy elderly, such as transit time, stool characteristics, and colonic environment. We did not find scFOS ingestion changed faecal weight and oro-fecal transit time in elderly. Gibson *et al*. have shown that prebiotics can increase stool output: they studied 8 volunteers under controlled diet, and showed that with 15 g/d fructo-oligosaccharides, stool output significantly increased from 136 to 154 g/d [27]. Other two human studies did not demonstrate increasing stool output [2,30]. but the diet was not controlled in none of these studies, which may have hidden any slight effect. In the study of Alles *et al*., 12 healthy subjects were given 4.8–19.2 g/d oligomate (52% galacto-oligosaccharides), which did not result in any change in bowel habit. However, the subjects started with unusually high faecal weights under controlled diet, 272 ± 26 g/d On the other hand, studies using probiotics demonstrated bifidobacteria could reduce human colonic transit time, but not all bifidobacteria strains have the same effects [31]. This specific strain-dependent effect could explain the reason why our prebiotic, which stimulates global endogenous bifidobacteria, had no effect.

In our study, the microbial transformation of cholesterol into coprostanol was not influenced by scFOS ingestion. Another study observed that sterol and fatty acid biohydrogenation by intestinal microflora is altered by oligosaccharide fermentation [32]. Coprostanol production results from intestinal anaerobic bacteria action [33]. Concerning bile acid metabolism, no differences were observed during the three periods. Furthermore, the use of poorly digestible carbohydrates in rats, hamsters and pigs demonstrated that prevention of microbial conversion of bile acids depended on the carbohydrate dose in the diet [23,34] This suggests that the low carbohydrate dose, 8 g/d scFOS, used in this experiment is unable to modify microbial conversion of bile acids.

Endogenous or exogenous bile acids, as well as dietary cholesterol are carcinogenic factors involved in colon cancer in laboratory animals [35,36] Various epidemiological studies suggest those steroids could also be involved in colon cancer in men [12,37]. According to these studies, low scFOS dose ingestion by humans, which prevented microbial conversion of cholesterol into cytotoxic molecule, (coprostanol, potentially carcinogenetic), could be interesting for humans. In our study, the intake of 8 g/d scFOS led to increasing faecal cholesterol. The mechanism of such increase could be related to decreasing cholesterol bacterial transformation, although we failed to find any significant sc-FOS effect on cholesterol bacterial metabolism. Moreover, the low scFOS dose used in our study was also probably not sufficient to significantly reduce microbial conversion of bile acids. However, in our previous study evaluating a higher scFOS dose (12.5 g/d), we also failed to show any significant effect in bile acids and neutral sterol [28]. These negative results could be explained by a questionable capacity of various bifidobacteria to take up cholesterol into their cellular membrane [38].

## Conclusion

Overall, we showed that 8 g/d scFOS ingested are well tolerated and led to significant increase in faecal bifidobacteria in healthy elderly. Under our experimental conditions, i.e. 8 g/d for 12 days, we failed to show any sc-FOS effects on OFTT, which is commonly increased in elderly living in industrialised countries. We found significant change in cholesterol metabolism, which could potentially exert protective action against colon cancer; however, this finding warrants further studies.

## List of abbreviations used

OFTT: Oro-fecal transit time; scFOS: Short-chain fructo-oligosaccharides

## Competing interests

The author(s) declare that they have no competing interests.

## Authors' contributions

YB participated in the study design, data collection, data analysis, and manuscript writing. LA participated in data collection. MR carried out bile acids and neutral sterols analysis. DP participated in data analysis and manuscript writing. FB designed the study and participated in manuscript writing. All authors read and approved the final manuscript.
